# Improved Outcomes and Therapy Longevity after Salvage Using a Novel Spinal Cord Stimulation System for Chronic Pain: Multicenter, Observational, European Case Series

**DOI:** 10.3390/jcm13041079

**Published:** 2024-02-14

**Authors:** Philippe Rigoard, Maxime Billot, Renaud Bougeard, Jose Emilio Llopis, Sylvie Raoul, Georgios Matis, Jan Vesper, Hayat Belaïd

**Affiliations:** 1 PRISMATICS Lab, Poitiers University Hospital, 86021 Poitiers, France; maxime.billot@chu-poitiers.fr; 2 Clinique de la Sauvegarde, 69009 Lyon, France; dr.bougeard@renaudbougeard.fr; 3 Hospital Universitario de la Ribera, 46600 Alzira, Valencia, Spain; llopis_joscal@gva.es; 4 CHU de Nantes-Hopital Laennec, 44800 Saint-Herblain, France; sylvie.raoul@chu-nantes.fr; 5 Uniklinik Köln, 50937 Köln, Germany; georgios.matis@uk-koeln.de; 6 Universitaetsklinikum Dusseldorf, 40225 Dusseldorf, Germany; jan.vesper@med.uni-duesseldorf.de; 7 Fondation Adolphe de Rothschild, 75019 Paris, France; hbelaid@for.paris

**Keywords:** chronic pain, spinal cord stimulation, system conversion, waveform therapy

## Abstract

Spinal cord stimulation (SCS) is proven to effectively relieve chronic neuropathic pain. However, some implanted patients may face loss of efficacy (LoE) over time, and conversion to more recent devices may rescue SCS therapy. Recent SCS systems offer novel stimulation capabilities, such as temporal modulation and spatial neural targeting, and can be used to replace previous neurostimulators without changing existing leads. Our multicenter, observational, consecutive case series investigated real-world clinical outcomes in previously implanted SCS patients who were converted to a new implantable pulse generator. Data from 58 patients in seven European centers were analyzed (total follow-up 7.0 years, including 1.4 years after conversion). In the Rescue (LoE) subgroup (*n* = 51), the responder rate was 58.5% at the last follow-up, and overall pain scores (numerical rating scale) had decreased from 7.3 ± 1.7 with the previous SCS system to 3.5 ± 2.5 (*p* < 0.0001). Patients who converted for improved battery longevity (*n* = 7) had their pain scores sustained below 3/10 with their new neurostimulator. Waveform preferences were diverse and patient dependent (34.4% standard rate; 44.8% sub-perception modalities; 20.7% combination therapy). Our results suggest that patients who experience LoE over time may benefit from upgrading to a more versatile SCS system.

## 1. Introduction

Chronic pain is a distressing condition, thought to affect around one-quarter of people worldwide [1], and is a leading cause of disability and disease burden. Low back pain is one of the top 10 contributors to years lived with disability in adults [2], impacting psychological and social conditions [3,4,5]. Since its first application in the late 1960s [6], spinal cord stimulation (SCS) using conventional paresthesia-based stimulation has proven to be an effective and efficient therapy for chronic low back and/or leg pain [7,8,9,10]. New SCS paradigms have been developed over the last 15 years, introducing neural-targeting algorithms, sub-perception therapies, and waveform combination capabilities supported by substantial clinical evidence [11,12,13,14,15,16,17,18,19,20].

While significant benefits from SCS therapy are sustained in the long term in most patients, some may become suboptimal over time and face loss of efficacy (LoE) [21,22,23,24]. LoE can occur when pain coverage is lost (i.e., with new onset pain or when stimulation is no longer perceived in the previous area [21,25]) or when patients have suboptimal pain relief despite no loss of coverage, implying stimulation tolerance that can affect up to one-third of patients in the long term [22,23]. The pathophysiology of stimulation tolerance is not yet fully understood but may include neural plasticity or fibrosis around the lead [21].

Once all of the potential device-related causes of LoE (e.g., lead migration, lead fracture, battery depletion, etc.) have been excluded and/or managed accordingly, several rescue strategies can be implemented. The objective of rescue therapy is to regain and sustain clinically significant pain relief and thus expand the durability of SCS therapy and avoid the need for explanting the SCS system [26,27,28]. If the implanted device is capable of delivering at least one alternative SCS modality, non-invasive reprogramming strategies can be useful to rescue LoE [16,29,30,31,32] and should be conducted first. However, the lack of programming capabilities in previous generations of implantable pulse generators (IPG) able to deliver only one stimulation modality may limit the possibilities for sustained pain relief.

Yet, it is possible to use a more versatile SCS device with advanced programming capabilities that provide full access to a wide range of therapeutic options. These modalities include supra- and sub-perception stimulation therapies that can be used either in isolation or in combination and enable the use of advanced temporal and spatial neural-targeting algorithms (e.g., customized field shapes using multiple independent current control, MICC) [14,15,16,17,18,20,33,34]. Simple, minimally invasive replacement of the IPG, using an adapter or not, can be performed, enabling access to multiple programmable solutions that allow stimulation to be tailored and adjusted over time. This ability could potentially overcome tolerance and avoid the need for explantation [34]. Several monocentric clinical studies have reported promising results after LoE patients were offered IPG conversion procedures, resulting in improvements in pain intensity, functional disability, and quality of life [34], as well as successful rescue of 78% of patients who then sustained significant benefits for up to one year after conversion [35].

Besides LoE, other patients with older-generation SCS devices may face suboptimal battery longevity and/or charging inconvenience. These patients may also benefit from an upgrade to more recent battery technology, which could expand the IPG’s longevity and simplify their charging experience.

Our objective in this multicenter, European study was to investigate real-world clinical outcomes in previously implanted SCS patients who converted to a multimodal SCS IPG offering multiple waveform options. We hypothesized that patients who converted to a newer system would report an improvement in overall pain scores that would be sustained in the long term.

## 2. Materials and Methods

### 2.1. Study Design

These are the initial results from a retrospective review of data obtained from de-identified patient records from a consecutive case series performed in seven centers throughout Europe. Ethics Committee approval was obtained from each site, and the study was conducted in accordance with Good Clinical Practice (ISO14155) guidelines and the Declaration of Helsinki. All patients provided written, informed consent, as required per local regulatory authorities.

### 2.2. Study Setting and Participants

Consecutive chronic pain patients (aged ≥ 18 years) who had been converted, via a direct lead connection or with the use of an adapter to a new SCS device (Boston Scientific Neuromodulation, Valencia, CA, USA) after they had received SCS therapy with a previously implanted system (any manufacturer, apart from Boston Scientific) were included. IPG conversion procedures were conducted between April 2016 and June 2022.

Each center applied its standard practice to decide whether to convert the patient’s existing IPG. The reasons for replacing the previous IPG with a different technology varied. In most cases, decisions to convert the previous device were motivated by suboptimal pain relief (patients experiencing moderate to severe pain and/or <50% pain relief with the previous device), reprogramming limitations (no alternative waveforms with the previous device), and/or longevity or charging issues. Two subgroups were further defined to differentiate patients who had a conversion procedure to restore the efficacy of SCS therapy (“Rescue” group) from those who were converted to a new IPG for a better experience with device longevity and/or charging (“Sustain” group).

There were no exclusion criteria, as per the study protocol. All patients eligible for SCS whose indications were compliant with the new device’s “directions for use” labeling and with local regulations were included in the study.

Data collection was organized by the center and consisted of reporting documented outcomes from patients’ medical files as they had been evaluated per standard of care. As a result, the type of clinical evaluations and the number and timing of follow-up visits could vary across sites and patients.

### 2.3. IPG Conversion

Patients who were previously implanted with SCS systems from multiple manufacturers had their IPG replaced with a multimodal Boston Scientific IPG (Spectra Wavewriter, Precision Spectra, Wavewriter Alpha, Precision Montage, Precision Novi, or Precision Plus) using an implantable adapter if needed (Precision M8 for Medtronic leads, Precision S8 for Abbott leads). For Nevro leads, a direct connection to the new IPG was possible and performed without using any adapter. In all patients, SCS leads from the previously implanted SCS system were kept in place. The conversion procedure consisted of performing a cutaneous incision at the level of the IPG pocket to remove the previous neurostimulator and connecting the new one to the implanted lead or extension.

The multiple independent current control (MICC) technology and customized algorithms embedded in the new SCS system were used to tailor stimulation programming, including adapting the shape of the electrical field to optimize spatial neural targeting and adjusting the temporal resolution of the signal using one or several waveform(s). The programming capabilities offered by the new IPG included one or more of the following SCS modalities:

- MICC-tonic SCS: supra-perception, paresthesia-based SCS modality that uses MICC technology and the Illumina 3D^TM^ programming algorithm (Boston Scientific). Illumina 3D^TM^ is a proprietary, neural-targeting algorithm that takes into account the 3D anatomical environment around the SCS leads to compute the electrical field that will best engage specific dorsal column fibers and cover the desired pain areas.
- Customized burst SCS (Burst 3D or MicroBurst 3D, Boston Scientific): sub-perception SCS modality delivering packets of burst stimuli in a regular manner. Burst stimulation leverages the Illumina 3D^TM^ algorithm to target the stimulation area and offers various settings (e.g., intra-burst frequency, inter-burst frequency, pulse width, number of pulses, etc.) that help to personalize the waveform to each patient.
- High-frequency/dorsal horn modulation (DHM) SCS: sub-perception SCS modality using high-frequency (≤1.2 kHz) stimulation and MICC and/or the Illumina 3D algorithm. High-frequency SCS can either use a focal target or a broad uniform field of stimulation using the Contour algorithm (Boston Scientific). High-frequency SCS has been shown to significantly reduce the wide dynamic range output [36], and the Contour algorithm implements a stimulation field designed to preferentially modulate the dorsal horn inhibitory interneurons [18,37].
- Fast-acting sub-perception SCS therapy (FAST) enables rapid onset of analgesia that combines precise placement of the stimulating electric field and precise dosing of a biphasic symmetric waveform at low frequency in a manner intended to engage surround inhibition for pain relief [38,39]. FAST therapy is programmed with the proprietary Illumina 3D^TM^ algorithm and uses a 90 Hz active recharge waveform to achieve 100% coverage before reducing the amplitude to a sub-perception level.
- Combination SCS therapy allows multiple waveforms to be layered in a simultaneous or sequential manner to engage various modalities and mechanisms of action. For example, MICC-tonic SCS could be simultaneously delivered with Contour SCS to produce both dorsal column activation and dorsal horn modulation to optimize pain relief.

### 2.4. Outcome Measures

All data were collected by the sites and their medical staff, as per standard practice and without sponsor involvement. Patient assessments were made before any SCS system was implanted, as well as prior to (pre-conversion) and immediately after implantation of the new SCS system (immediate post-conversion follow-up), and at the latest available follow-up (last follow-up). Demographic information was recorded, along with pain location, surgical history, and reason for conversion. Pain intensity was evaluated using the numerical rating scale (NRS, scored from 0 = no pain to 10 = worst pain; a score ≤3 corresponds to mild pain, 4–6 to moderate pain, and ≥7 to severe pain [40]). Patient preference for a programming modality was also recorded. The Oswestry Disability Index (ODI; 0 = no disability to 100 = highest level of disability) for assessing functional disability and responder rates (number of patients with at least 50% reduction in pain scores) were calculated for the “Rescue” subgroup.

Due to the retrospective design of this study, study outcomes reflect the clinical evaluations that were documented by the sites, as per their standard practice, and the available data were analyzed from only those patients who had completed follow-up at the time of the data snapshot. As such, the number of patients assessed fluctuated over time.

### 2.5. Statistical Analysis

A Kolmogorov–Smirnov test was performed to confirm the normality of the change in NRS score. For demographic data and NRS scores, means and standard deviations were determined for the Overall group of patients, as well as for the “Rescue” and “Sustain” subgroups. Descriptive analysis was used for the responder rates, which were calculated based on individual NRS pain scores before and after IPG conversion. A paired *t*-test with a two-sided 0.05 significance level was used to calculate whether the mean reduction in pre-conversion baseline pain was greater than 0. For the statistical procedure measuring overall NRS changes over time in both the Overall group and the Rescue subgroup, the Mixed Effect Model was used with three time points (baseline, post-conversion immediate follow-up, and last follow-up). Continuous variables are presented as mean ± standard deviation, while categorical variables are presented by frequency and percentage. All statistical analyses were performed using SAS System Version 9.3 software or above (SAS Institute Inc., Cary, NC, USA). The missing data were not imputed.

## 3. Results

### 3.1. Patient Population

Fifty-eight eligible patients (mean age 58.3 ± 9.5 years, 46.5% females) were included in the analysis. Patients suffered from pain in their low back and/or legs (Table 1). Prior to any SCS implant, the mean overall pain score (NRS) was 7.8 ± 1.9. At the time of conversion, patients had been treated with spinal cord stimulation for a mean of 5.6 ± 4.1 years.

**Figure 1:**
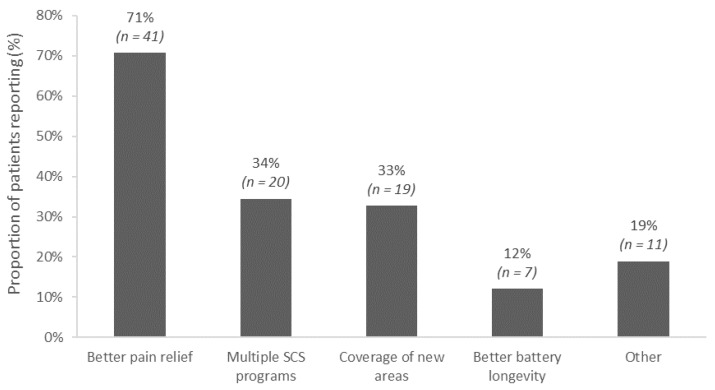
Reasons for converting to the new spinal cord stimulation implantable pulse generator (N = 58). Multiple reasons could be selected by each patient.

Treatment goals and expectations differed depending on the motivations for converting to a different IPG. The most frequent reasons patients chose to convert to a new SCS system were to improve pain relief (71%), to obtain access to multiple stimulation modalities (34%), for coverage of new pain areas (33%), and/or for better battery longevity (12%). Some patients reported multiple reasons (Figure 1).

Two subgroups were further defined to delineate the outcomes in converted patients based on their pre-conversion pain scores and reasons for conversion:

- Rescue of LoE (Rescue group): patients who had moderate to severe pain based on pre-conversion overall pain scores (NRS ≥ 4/10) or those who chose to convert for any one of the following reasons: better pain relief, access to multiple stimulation modalities, or coverage of new pain areas (*n* = 51).
- Sustain group: patients who had mild pain based on their pre-conversion overall pain score (NRS ≤ 3/10) or who chose to convert for better battery longevity (*n* = 7).

The overall average pre-conversion pain score (NRS) was 7.3 ± 1.7 in the Rescue group (*n* = 49) and 1.5 ± 1.2 in the Sustain group (*n* = 7).

### 3.2. Conversion Procedure

In all patients, SCS leads/extensions from the previous implanted system remained in place. In all patients but five (8.6%), adaptors were used to connect the leads/extensions to the new IPG (Table 2). Spectra Wavewriter was implanted in the majority of patients (*n* = 29, 50.0%), followed by Wavewriter Alpha (*n* = 12, 20.7%).

### 3.3. Post-Conversion Clinical Outcomes

#### 3.3.1. All Patients

**Figure 2:**
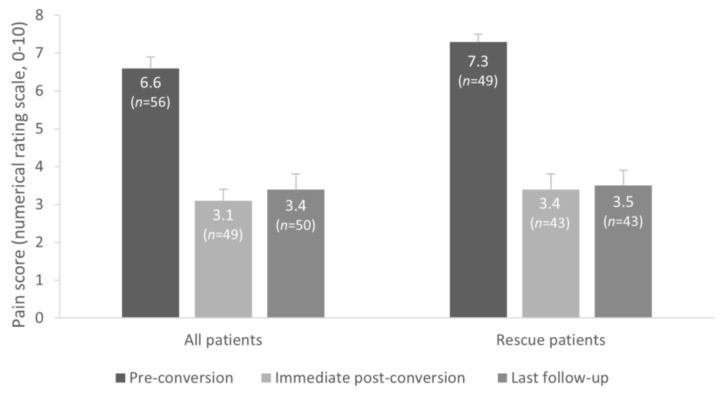
Overall NRS (0–10) pain scores (mean ± standard error) from the pre-conversion baseline to the immediate post-conversion and last follow-up evaluations (mean 1.4 years after new IPG implant) in the Overall and Rescue groups.

The Overall group of patients reported an average pre-conversion pain score of 6.6 ± 2.5 (*n* = 56) with the previous system post-optimization. Following the IPG upgrade procedure, the overall NRS pain score significantly decreased to a level of 3.1 ± 2.4 (*n* = 49, *p* < 0.0001) and was sustained until the last follow-up, i.e., 1.4 years after conversion (mean NRS 3.4 ± 2.5, *n* = 50, *p* < 0.0001) (Figure 2). With their new SCS therapy, patients experienced a significant and sustained reduction in their NRS score when compared to the level of their pain with the previous system.

#### 3.3.2. Rescue (LoE) Subgroup

In the Rescue (LoE) subgroup (*n* = 51), the mean pre-conversion overall pain score was 7.3 ± 1.7, despite programming optimization, and close to the level of pain reported by these patients before they started SCS therapy (7.8 ± 1.9).

After their previous device was replaced with the new IPG, patients reported a significant improvement in overall pain compared to pre-conversion (mean 4.1 ± 2.8-point reduction in the NRS score, *p* < 0.0001), with NRS pain score decreasing from 7.3 ± 1.7 before IPG was replaced to 3.4 ± 2.4 immediately after conversion (*p* < 0.0001). The improvement with the new SCS system was sustained at the last follow-up (mean NRS score 3.5 ± 2.5, *p* < 0.0001) (Figure 2).

The responder rate (proportion of patients reporting 50% pain relief or more) immediately after conversion was 78.0% (*n* = 32/41) and 58.5% (*n* = 24/41) at the last follow-up (1.4 years on average after conversion). Furthermore, 48.8% (*n* = 20/41) and 39.0% (16/41) of the Rescue patients reported ≥70% decrease in overall pain after conversion and at the last follow-up, respectively.

There was also a significant improvement in patients’ disability, with a mean reduction of 18.5 points in the ODI scores when comparing the pre-conversion status (63.9 ± 14.4, *n* = 14) to the last follow-up evaluations (40.8 ± 18.8, *n* = 23) (*p* = 0.01).

#### 3.3.3. Sustain Subgroup

In patients for whom the conversion was solely to benefit from a higher battery longevity or better charging experience (*n* = 7), the average pre-conversion pain score was 1.5 ± 1.2 and remained below 3/10 until the last follow-up (1.3 years after the new IPG was implanted).

#### 3.3.4. Waveforms Usage

**Figure 3:**
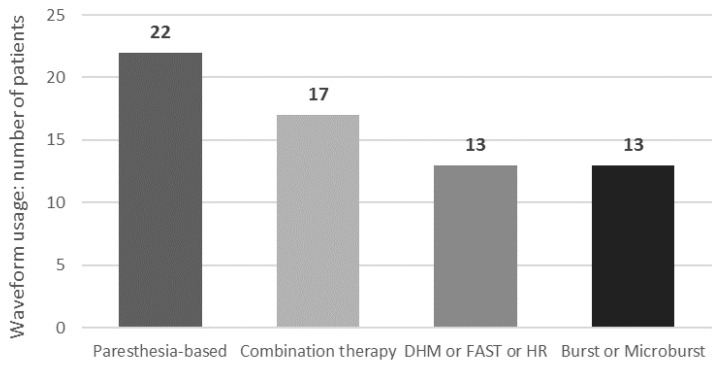
Waveform usage at last follow-up. Multiple waveforms may be used by each patient. DHM: dorsal horn modulation; FAST: fast-acting sub-perception; HR: high-rate SCS.

Patients reported their SCS program usage following conversion. At the last follow-up, the “MICC-paresthesia based SCS” modality was used the most, followed by combination SCS, then sub-perception therapies (burst/microburst or high-rate/DHM/FAST) (Figure 3). Patients could report the use of multiple programs and adjust their therapy as needed using their remote control.

## 4. Discussion

Our multicenter, observational, consecutive case series demonstrated that patients who converted to a new SCS system reported a significant improvement in overall pain scores that was sustained for 1.4 years post conversion. The majority of patients (88%, *n* = 51/58) were offered an IPG conversion procedure due to the loss of efficacy they faced with their previous system despite programming optimization. These findings support our hypothesis that new IPG with the capability to deliver multiple stimulation modalities and programming options can help restore SCS efficacy and that undertaking a conversion procedure may prevent the need for future explantation.

It is now well established that some patients using SCS for chronic pain may see their therapeutic response decrease (i.e., LoE) several years after their initial implant and may become totally refractory to SCS treatment [21,22,23,24]. Multiple clinical reports have described LoE cases and shown that 12–68% of patients became refractory to their initial SCS treatment after a period of 2–4.0 years [21,23,24,41]. LoE can have serious consequences, and multiple real-world reports have demonstrated that the primary reason for explants was inadequate pain relief [26,27,28]. Three large patient cohorts estimated that 41–52% of SCS explants in the long term were due to LoE [26,27,28], while 81% of patients from a cohort of 129 patients who underwent explantation of their SCS system over a nine-year period gave LoE as the primary reason [42].

The explantation rate due to inadequate pain relief is reported to be lower when using multimodal devices (2.4% [43]) compared with traditional SCS systems (around 10% [27,44]), possibly due to the ability to easily switch programs when pain relief is no longer sufficient or when the pain condition evolves with time [31]. Most of the systems used in early studies assessing real-world, long-term outcomes in patients experiencing LoE were non-versatile and had limited reprogramming capabilities, possibly compromising their ability to rescue LoE patients with the existing IPG and thus increasing the need for explantation. In 2014, Deer et al. [22] described “stimulation tolerance” as a difficult-to-predict “biologic complication” of SCS that could occur during the course of patient follow-up. The recommendations from the Neuromodulation Appropriateness Consensus Committee (NACC) group to help prevent or alleviate stimulation tolerance included the use of more versatile IPGs, which could “offer the possibility of choosing between paresthesia and paresthesia-free stimulation and modulation capabilities” [22]. Since then, various clinical reports have documented variable rates of success in patients using standard-rate SCS who experienced LoE and were subsequently converted to a system offering different modalities such as high-density SCS [29], BurstDR stimulation [24,30], 10 kHz SCS [45,46], or system with versatile capability [34,35]. In all of these experiences, the failed therapy was conventional standard-rate SCS therapy using single-source technology; however, it has been shown that LoE can also occur with other modalities [41]. Results from the WHISPER randomized controlled trial (RCT) demonstrated that a device capable of providing multiple neurostimulation therapies provided superior long-term outcomes when subjects were able to choose the most effective therapy [47]. In the MULTIWAVE crossover RCT, the responder rate increased by up to 25% when a device capable of such versatility was used [20,33], with a responder rate of 95% considering multidimensional index assessment [48]. Our own results demonstrated that in patients with LoE who converted to a new IPG (after more than five years of successful treatment with their previous SCS system), the mean NRS pain score decreased by 4.1 points (*p* < 0.0001) compared to pre-conversion, with a treatment responder rate of 58.5% (≥50% improvement in overall pain) at the last follow-up. In addition, disability also improved after conversion in these patients, as illustrated by the 18.5-point clinically significant reduction in the ODI score. Our results are consistent with previous reports of rescue experiences using similar devices [34,35], which have found that 12 months after conversion, pain scores were reduced by 4.4 points and 4.6 points, respectively, and ODI improved by 13.7 points [34]. The “sustain” subgroup of patients, although limited in size (*n* = 7), maintained the efficacy of SCS with their new device for up to 1.3 years after conversion. Previous studies had reported that SCS efficacy can be sustained over time after replacing the IPG, and that pain relief after replacement did not differ when compared to de novo implants [49,50].

Interestingly, we found that a significant number of patients (N = 22) used the MICC-tonic SCS modality as part of their rescue therapy, suggesting that spatial neural targeting may be an important factor to consider when optimizing standard-rate SCS and may play a role in overcoming lead fibrosis issues. Indeed, a study in chronic low back pain has demonstrated that SCS using 3D neural targeting led to better long-term pain relief over two years compared to conventional SCS, regardless of pain location [14]. Another finding derived from our evaluation was that the improvement in efficacy observed following conversion, in contrast to the use of monotherapy that precipitated the subsequent LoE, did not appear to be dependent on a particular preference for a specific rescue waveform. After conversion, 37.9% of patients used standard-rate paresthesia-based MICC SCS therapy, 44.8% used one of the various sub-perception modalities now available on their device, while 29.3% used combination SCS therapy. These results suggest that device versatility and programming capabilities are likely important for achieving optimal and personalized responses within the highly diverse cohort of patients who experience LoE and are consistent with the findings of Andrade et al. [35] and Rigoard et al. [34]. Furthermore, sustaining the efficacy of SCS therapy for years after implantation is important for relieving the level of burden on patients and healthcare systems. In our study, LoE patients had already experienced 5.6 years of successful therapy with their initial SCS system. The IPG conversion procedure enabled them to regain that efficacy and prolong the benefits of therapy for an additional mean of 1.4 years to date, resulting in almost 7 years of significant pain relief when using SCS therapy. In fact, the cumulative, real-world data collected over such a long time period in consecutive patients are a strength of our multicenter, international study and confirm that adaptable SCS therapy in well-monitored patients can provide effective, long-term pain relief.

Our study does have some limitations. Due to the retrospective nature of the study, the analysis is limited to only those data points that are available based on documented medical chart review per standard of care, without protocol, constant time points, and standardized outcomes. Therefore, some data are missing, and a limited number of multidimensional assessments were reported. There was also an imbalance in the number of patients in the Rescue and Sustain subgroups. Although our findings demonstrate increased and sustained efficacy in patients who experience LoE, it is necessary to confirm these data in larger and/or controlled studies and to further analyze the impact of flexible SCS therapy in patients who have already achieved relatively good pain relief. While a 1.4-year follow-up could be considered a strength of our study, offering pain relief in LoE patients, longer follow-ups are needed to capture the potential return of LoE with the new device. Despite the positive outcomes that we observed, future research is needed to obtain a greater understanding of the causes and mechanisms of LoE and to more precisely characterize the clinical profiles of LoE patients and the evolutions in their pathology that could explain why they became refractory to SCS. More data (e.g., large samples of patients, multiple datapoints) and artificial intelligence algorithms may ultimately enable better prediction and personalization of the neurostimulative modality(-ies) utilized by patients in the context of LoE and possibly help to prevent or reduce the incidence of LoE. Finally, the characteristics between the previous and new IPGs were not collected in our study. These elements, such as MRI compatibility, should be documented to ensure, at the very least, a similar capability of the systems. Improvements in MRI conditional compatibility of such hybrid SCS systems need to be further developed in the future.

## 5. Conclusions

Our clinical evaluation demonstrates that a simple conversion procedure was able to salvage SCS therapy and extend therapy longevity in chronic pain patients experiencing loss of efficacy. The level of pain reduction achieved following conversion was maintained in the long term (mean 1.4 years to date). Prospective randomized controlled trials are now needed to further confirm these findings.

## Figures and Tables

**Table 1:** Patient characteristics (*n* = 58).

Characteristics	Patients
Sex—female, *n* (%)	27 (46.5)
Age (years), mean ± SD	58.3 ± 9.5, *n* = 52
Pain location prior to IPG conversion, *n* (%) (multiple locations may be reported)	Low back/legs, 33 (57.0) Lower limbs, 25 (43.1)
Pain prior to any SCS implant, mean ± SD	7.8 ± 1.9, *n* = 47
Pain prior to IPG conversion, mean ± SD ALL patients Rescue group Sustain group	6.6 ± 2.5, *n* = 56 7.3 ± 1.7, *n* = 49 1.5 ± 1.2, *n* = 7
Follow-up duration (years), mean ± SD [range in years] With previous IPG With new IPG	5.6 ± 4.1 [0.02–8.25], *n* = 58 1.4 ± 1.4 [0.04–18.98], *n* = 50
Waveform used priori conversion	
Paresthesia-based	*n* = 39
Paresthesia-free	*n* = 15

IPG, implantable pulse generator; SCS, spinal cord stimulation; SD, standard deviation.

**Table 2:** Device-related information (N = 58).

Device-Related Information	Patients
Patients prior to conversion, type of adaptors used *n* (%) M8/M1 adaptor S8 adaptor No adaptor	44 (75.9) 9 (15.5) 5 (8.6)
Patients after conversion, type of IPG implanted, *n* (%) Spectra Wavewriter Wavewriter Alpha Precision Spectra Precision Montage Precision Novi Precision Plus Not reported	29 (50.0) 12 (20.7) 11 (18.9) 3 (5.2) 1 (1.7) 1 (1.7) 1 (1.7)

## Data Availability

The data, analytic methods, and study materials for this clinical study will be made available to other researchers in accordance with the Boston Scientific Data Sharing Policy: https://www.bostonscientific.com (accessed on 5 February 2024).
